# Photosynthetic Responses of Forests to Elevated CO_2_: A Cross-Scale Constraint Framework and a Roadmap for a Multi-Stressor World

**DOI:** 10.3390/biology14111534

**Published:** 2025-11-01

**Authors:** Nan Xu, Tiane Wang, Yuan Wang, Juexian Dong, Wenhui Bao

**Affiliations:** 1 Key Laboratory of Heilongjiang Province for Cold-Regions Wetlands Ecology and Environment Research, Harbin University, Harbin 150086, China; xunan0451@126.com (N.X.); harbinwte@126.com (T.W.); wy20012021@163.com (Y.W.); 13078783263@163.com (J.D.); 2 College of Architecture and Energy Engineering, Wenzhou University of Technology, Wenzhou 325011, China

**Keywords:** elevated CO_2_, forest ecosystems, photosynthesis, CO_2_ fertilization constraint, photosynthetic acclimation, nutrient limitation, multi-stressor interactions, signal transduction, gene function, FACE experiments, carbon cycle, climate change

## Abstract

**Simple Summary:**

Forests store carbon and so are often seen as a simple fix for climate change. In reality, their growth under higher carbon dioxide is limited by several everyday needs. This review follows carbon dioxide from the air into a leaf and then through the whole forest. The first step—the leaf’s sugar-making process—can rise for a while, but plants soon adjust and the boost fades. The next hurdle is food for plants: nutrients such as nitrogen and phosphorus. Without enough of these, extra carbon dioxide is like pressing the accelerator with an empty tank. Water and heat also matter, as do choices plants “make” about whether to build leaves, wood, or roots. Looking across many field trials and computer studies, we find that nutrient limits are the main brake on long-term gains. This matters for society because climate plans should not assume large, lasting growth everywhere. Targeting restoration on fertile soils and improving models to include nutrients and water can deliver more reliable climate benefits.

**Abstract:**

Rising atmospheric CO_2_ is expected to fertilize forest photosynthesis; yet, ecosystem-scale observations often reveal muted responses, creating a critical knowledge gap in global climate projections. In this review, we explore this paradox by moving beyond the traditional ‘CO_2_ fertilization’ paradigm. We propose an integrated framework that positions elevated CO_2_ as a complex modulator whose net effect is determined by a hierarchy of cross-scale constraints. At the plant level, photosynthetic acclimation acts as a universal first brake on the initial biochemical potential. At the ecosystem level, nutrient availability—primarily nitrogen in temperate/boreal systems and phosphorus in the tropics—emerges as the dominant bottleneck limiting long-term productivity gains. Furthermore, interactions with the water cycle, such as increased water-use efficiency, create state-dependent dynamic responses. By synthesizing evidence from pivotal Free-Air CO_2_ Enrichment (FACE) experiments, we systematically evaluate these constraining factors. We conclude that accurately predicting the future of the forest carbon sink necessitates a paradigm shift: from single-factor analysis to multi-stressor approaches, and from ecosystem-scale observations to an integrated understanding that links these phenomena to their underlying molecular and genetic mechanisms. This review provides a roadmap for future research and informs more realistic strategies for forest management and climate mitigation in a high-CO_2_ world.

## 1. Introduction

Forests play a pivotal role in the global carbon cycle, sequestering a significant portion of anthropogenic CO_2_ emissions primarily through photosynthesis [1,2,3,4,5,6]. As atmospheric CO_2_ concentrations continue their unprecedented rise, understanding the response of this fundamental process is paramount for predicting future climate trajectories and ecosystem service provisioning [7,8]. At the leaf level, the direct effects of elevated CO_2_ on C_3_ plants are widely observed: increased substrate for Rubisco and reduced photorespiration promise a substantial boost in photosynthetic efficiency [9,10]. In this review, the term “multi-stressor world” refers to the interactive context in which rising atmospheric CO_2_ coincides with other major global change drivers, including warming, drought and vapor-pressure deficit (VPD), tropospheric ozone, nitrogen deposition, phosphorus limitation, and disturbance regimes such as fire.

However, a crucial scientific paradox emerges when scaling from the leaf to the globe. The large potential photosynthetic enhancement of 30–60% observed in short-term, controlled studies [9,11,12,13,14] starkly contrasts with the more modest and highly variable productivity gains (often 0–25%) documented in long-term, ecosystem-scale experiments and forest inventories [15,16,17,18]. This discrepancy suggests the existence of powerful constraining mechanisms that operate across multiple biological and temporal scales [11,12,19,20,21]. Ignoring these constraints leads to overly optimistic projections of the terrestrial carbon sink, a critical error for climate policy [22].

This review systematically deconstructs this paradox. We move beyond a simple inventory of CO_2_ effects to build an argument-driven synthesis. Our central thesis is that elevated CO_2_ acts less as a simple fertilizer and more as a complex ecosystem modulator, whose ultimate impact is determined by a hierarchy of interacting limitations. We will dissect these limitations, from plant-level physiological feedbacks to ecosystem-scale nutrient and water controls. By re-evaluating evidence from key experimental platforms like FACE [13,14], this review aims to: (1) establish a clear hierarchy of factors that constrain the CO_2_ fertilization effect; (2) use this framework to explain the divergent responses observed across major forest biomes; and (3) propose a path forward for future research that bridges the gap between ecosystem-scale observations and their underlying molecular and genetic mechanisms. Unlike previous studies that mainly relied on Progressive Nutrient Limitation (PNL) or source–sink imbalance theory, the “hierarchical constraint framework” proposed in this review extends existing concepts in two major ways. First, it emphasizes a continuous chain of constraints—from leaf biochemistry and plant-level physiological regulation to community structure and ecosystem nutrient cycling—and highlights the predictable temporal sequence among these limitations. Second, it integrates nitrogen/phosphorus limitation with other key modulators, including water-use efficiency (WUE), drought and vapor-pressure deficit (VPD), and carbon allocation/root–mycorrhizal interactions, thereby forming a unified multi-stressor response surface that can explain divergent responses across forest biomes.

## 2. The Biochemical Potential: Why We Expect a Strong CO_2_ Effect

**Figure 1:**
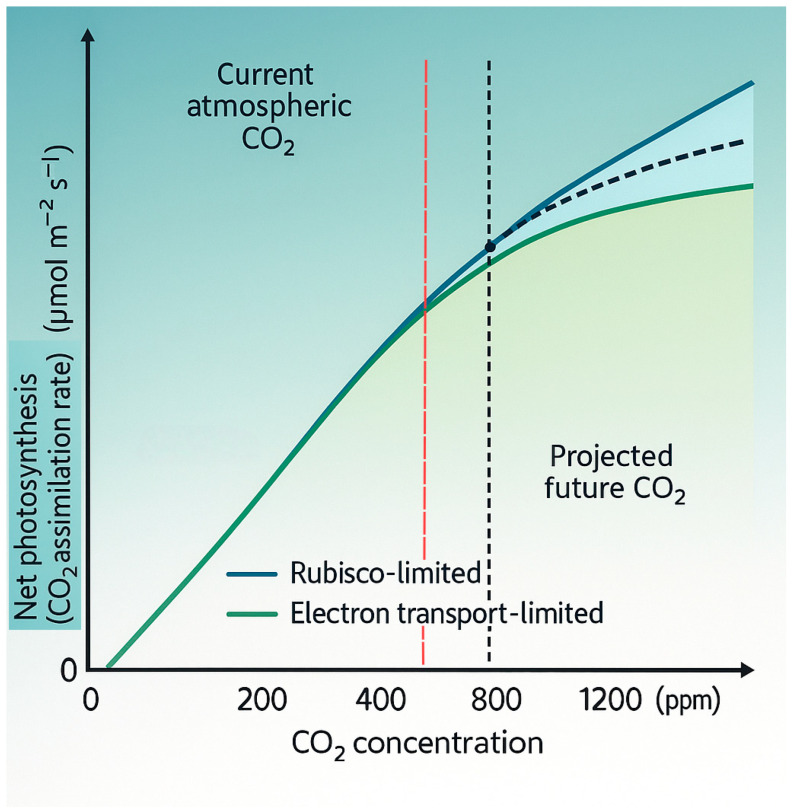
Theoretical A/C_i_ response of a C_3_ leaf under elevated CO_2_. The *y*-axis shows net CO_2_ assimilation rate (A, μmol m^−2^ s^−1^); the *x*-axis shows intercellular CO_2_ concentration (C_i_, μmol mol^−1^). Blue and green curves depict Rubisco-limited and RuBP-regeneration-limited segments, respectively; the dashed line denotes the realized rate bounded by the minimum of the two. Vertical lines mark ~420 and ~800 μmol mol^−1^. Assumptions: constant leaf temperature, no nutrient or water constraints, and parameterization from meta-analyses of C_3_ tree species. The curve illustrates a theoretical maximum that is typically attenuated by acclimation and ecosystem-scale constraints.

The direct physiological basis for the CO_2_ fertilization effect in C_3_ plants, which include virtually all tree species, is fundamentally sound and well documented. Elevated CO_2_ enhances leaf-level photosynthesis through two primary mechanisms. First, it increases the substrate (CO_2_) availability at the active site of the enzyme Rubisco, thereby boosting the carboxylation rate [23,24], especially under conditions where this process is the primary limiting step [23,24,25]. Second, by increasing the CO_2_:O_2_ ratio at the site of carboxylation, it competitively inhibits the oxygenase activity of Rubisco, a key reaction initiating photorespiration [26,27]. This reduction in photorespiration, an energy- and carbon-wasteful process, directly increases the net carbon gain of the leaf [28]. These combined effects, demonstrated consistently in short-term experiments, can lead to instantaneous photosynthetic rate increases of 30–60% at CO_2_ concentrations projected for the end of the century (~550–700 ppm) [19,20], establishing a high ceiling for the potential ecosystem response (Figure 1).

## 3. The First Attenuation: Photosynthetic Acclimation as an Inevitable Plant-Level Feedback

While the initial biochemical stimulation is potent, it is rarely sustained over the long term. The first and most universal brake on the CO_2_ effect is photosynthetic acclimation, a suite of physiological adjustments that downregulate photosynthetic capacity in response to prolonged exposure to elevated CO_2_ [11,12]. This process should not be viewed as a malfunction, but rather as an adaptive resource optimization strategy by the plant [29,30,31,32,33,34]. A primary driver for this acclimation is the potential imbalance between the rate of carbohydrate production (the “source”) and the plant’s ability to utilize or store these carbohydrates for growth and metabolism (the “sink”) [31,32,33,34,35,36,37]. When carbon assimilation outpaces sink capacity, the resulting accumulation of leaf sugars can trigger feedback inhibition, leading to the downregulation of genes related to photosynthesis and carbon fixation [11,38,39,40,41].

**Figure 2:**
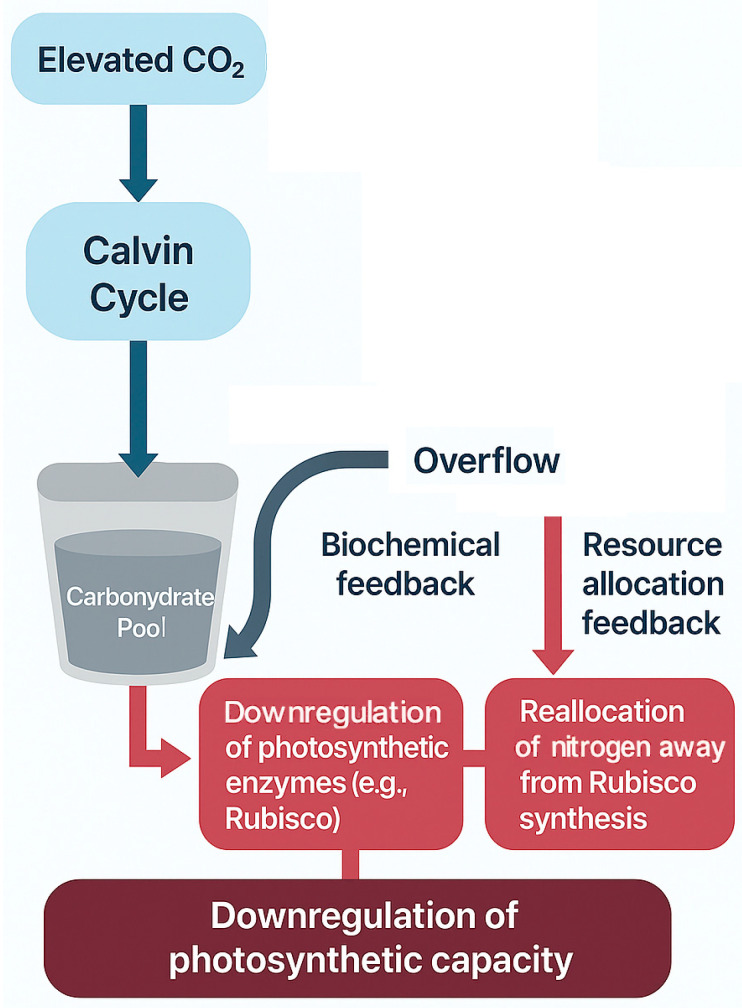
The First Attenuation: Key Feedback Mechanisms of Photosynthetic Acclimation. This schematic diagram illustrates the core feedback loops driving photosynthetic acclimation in plants under elevated CO_2_. (1) Elevated CO_2_ directly enhances the Calvin cycle, increasing carbohydrate production. (2) When the rate of carbohydrate production (source) exceeds the plant’s capacity for utilization in growth and storage (sink), an accumulation of sugars occurs. (3) This accumulation triggers two primary negative feedback regulations: (a) Biochemical feedback, which involves signal transduction pathways that directly inhibit the expression of photosynthesis-related genes and reduce the activity of the Rubisco enzyme; and (b) Resource allocation feedback, where the plant reallocates valuable resources, such as nitrogen, away from the photosynthetic machinery (e.g., Rubisco) to other components that are more limiting for growth. Together, these two feedback loops lead to a downregulation of photosynthetic capacity, thereby attenuating the long-term stimulatory effect of elevated CO_2_ on photosynthesis.

This feedback mechanism manifests through several key physiological changes. Most commonly, plants exhibit a decrease in the content and enzymatic activity of Rubisco (a reduction in the maximum carboxylation capacity, Vc_max_) [29,34,42,43,44,45]. This is often accompanied by a strategic reallocation of valuable nutrients, particularly nitrogen, away from the now less-limiting photosynthetic apparatus towards other functions that may be more limiting to overall growth [35,36,46,47,48]. The ratio between carboxylation capacity and electron transport capacity (Vc_max_:J_max_) may also shift, reflecting a holistic re-balancing of the plant’s internal resources under a new high-carbon reality [37,38,49,50,51]. Photosynthetic acclimation is also species-dependent, with different tree species showing variable adjustments in Vcmax, Jmax, and nitrogen reallocation under elevated CO_2_, as illustrated in Figure 2.

The strength and timing of photosynthetic acclimation are not uniform; they vary significantly depending on species, a plant’s sink strength (e.g., young, rapidly growing trees versus mature trees), nutrient availability, and other interacting environmental conditions [39,40,52,53,54,55]. Evidence from various Free-Air CO_2_ Enrichment (FACE) experiments provides clear empirical examples of these dynamics. For instance, sweetgum trees (Liquidambar styraciflua) at the Oak Ridge FACE site showed a notable 10–20% reduction in Vc_max_ over the course of the experiment, eroding the initial CO_2_-driven photosynthetic gains [41,56,57,58,59]. In contrast, loblolly pine (Pinus taeda) at the Duke FACE site, which had a very high sink demand, exhibited minimal photosynthetic acclimation, maintaining a sustained photosynthetic enhancement over many years [42,43,59,60,61]. These contrasting results underscore that while acclimation is a widespread phenomenon, its magnitude is context-dependent (Table 1).

Ultimately, photosynthetic acclimation represents the first process that creates the gap between the large biochemical potential and the modest whole-plant responses by down-regulating Vcmax and Jmax and reallocating nitrogen under sustained CO_2_ enrichment. It is an intrinsic, plant-level feedback that ensures the CO_2_ fertilization effect is already being attenuated before ever facing the even larger constraints imposed by the wider ecosystem [43,62,63,64,65].

## 4. The Dominant Constraint: Nutrient Limitation as the Primary Ecosystem Bottleneck

If photosynthetic acclimation is the first brake applied at the plant level, then nutrient availability is the unyielding roadblock that ultimately governs the magnitude and sustainability of the CO_2_ fertilization effect at the ecosystem scale. Forests are complex systems where productivity is co-limited by multiple resources [44,45,66,67,68]; simply increasing the carbon supply to a system that is starving for nitrogen (N) or phosphorus (P) is akin to pressing a car’s accelerator with the fuel tank empty. This principle, known as progressive nutrient limitation (PNL), has now emerged as the single most important explanation for the muted and often transient responses of mature forests to elevated CO_2_ [46,47,48,69,70,71]. The specific limiting nutrient, dictated by geology, climate, and ecosystem age, creates clear and predictable delineations in forest responses across the globe.

In many temperate and most boreal forests, nitrogen is the primary currency of growth and productivity. While elevated CO_2_ may initially stimulate N uptake to support enhanced growth, this increased demand can rapidly deplete the available soil N pools, thereby constraining any further growth response over time [35,49,50,51,52,72,73,74]. The Oak Ridge FACE experiment provided a classic, long-term demonstration of this process. An initial ~20% enhancement in net primary productivity (NPP) in the sweetgum plantation declined to just ~10% after several years, a trend directly attributed to the ecosystem’s inability to supply enough nitrogen to meet the CO_2_-driven demand [49]. This highlights that without a concurrent and sustained increase in N supply, any CO_2_-driven growth spurt in N-limited systems is destined to be short-lived. Similarly, the productivity of boreal forests, while also constrained by low temperatures and short growing seasons, is fundamentally limited by N-poor soils, which severely caps their potential response to rising CO_2_ [70] (Table 2).

In contrast, across vast expanses of the tropics and on geologically ancient soils in some temperate regions, phosphorus availability is the critical constraint [44,45,51,74]. This has led to one of the most striking and sobering results in the history of FACE research: at the EucFACE site in a P-limited Australian woodland, nearly a decade of CO_2_ enrichment produced no significant increase in NPP [53,54,75]. This powerful finding suggests that large swathes of the planet’s forests, particularly the vital tropical carbon sinks, may not respond positively to rising CO_2_ at all—a reality that many current carbon-cycle models still fail to capture adequately [52,56,57,76,77]. Indeed, emerging results from the AmazonFACE project and associated modeling studies increasingly confirm that P availability will be the ultimate arbiter of the Amazon basin’s response to future CO_2_ levels [55,76,78].

**Figure 3:**
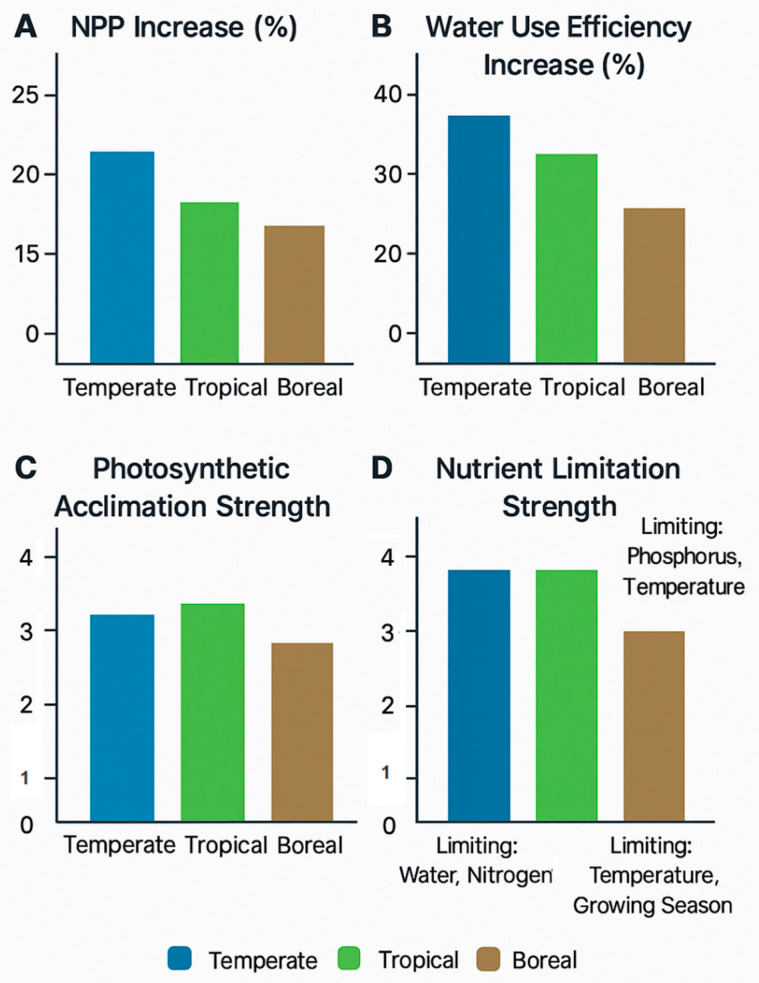
Biome-level comparison of constraints on photosynthetic responses to elevated CO_2_. Values are expressed as dimensionless indices normalized to the site-level mean under ambient CO_2_ (100% baseline). “Photosynthetic acclimation strength” and “nutrient limitation strength” are qualitative indices (1–5) synthesized from FACE and field studies (see Table 2 and Section 4 for details). Higher values denote stronger attenuation of the CO_2_ fertilization effect.

Therefore, the well-documented variation in responses among different forest types (Figure 3) is not an arbitrary collection of facts, but rather a predictable outcome dictated by their underlying biogeochemical context [55,56]. The “strong” response of the loblolly pine at the Duke FACE experiment (a sustained 23% NPP increase) was observed on a relatively fertile site where trees could effectively acquire sufficient nutrients to support enhanced growth [15,42,57,58,59,60,61,79,80]. This stands in stark contrast to the negligible response in P-limited systems like EucFACE, revealing a clear hierarchy of controls where nutrient availability can completely override the direct biochemical potential of CO_2_.

**Table 2:** Key Processes and Mediators of Nutrient Limitation under Elevated CO_2_.

Process	Description	Key References
Progressive nitrogen limitation	Gradual decrease in soil N availability constraining long-term CO_2_ response	[45,49]
Phosphorus limitation	Strong constraint on CO_2_ fertilization, especially in tropical forests	[50,53]
Mycorrhizal associations	Fungal symbioses mediating plant nutrient acquisition under elevated CO_2_	[72,81]
Root allocation changes	Increased carbon allocation to roots for enhanced nutrient acquisition	[52,72]
Soil organic matter dynamics	Changes in decomposition rates and soil carbon storage	[82,83]
Nutrient use efficiency	Adjustments in plant nutrient utilization strategies	[17,35]
Soil microbial activity	Altered microbial communities and functions affecting nutrient cycling	[52,72]

Note: This table summarizes the key nutrient cycling processes that constrain a sustained forest response to elevated CO_2_ at the ecosystem scale. These processes, particularly Progressive Nitrogen Limitation (PNL) and Phosphorus (P) Limitation, are central to explaining why the CO_2_ fertilization effect is substantially diminished in the real world, supporting the argument of Section 4 regarding the “primary ecosystem bottleneck”.

## 5. Cross-Scale Modulators: How Water, Climate, and Carbon Allocation Reshape the CO_2_ Response

Beyond the primary constraints of photosynthetic acclimation and nutrient limitation, the final ecosystem response to elevated CO_2_ is further shaped by a suite of interacting modulators. These factors do not act in isolation but rather create a complex, state-dependent response surface where the effect of CO_2_ can be either amplified or dampened depending on the prevailing conditions [22,56,84,85,86]. Understanding these interactions is critical for moving from a static view of CO_2_ effects to a dynamic and predictive understanding of forest function in a changing world.

One of the most consistent and well-documented physiological responses to elevated CO_2_ is the partial closure of leaf stomata, which reduces water loss via transpiration [9,57,58,59]. This directly increases leaf-level water use efficiency (WUE)—the amount of carbon gained per unit of water lost [15,16,60,61,62,63,87]. This enhancement of WUE is not merely a secondary benefit; it is a critical modulator that intimately links the carbon and water cycles. In water-limited ecosystems or during periods of drought, enhanced WUE can act as a powerful amplifier of the CO_2_ effect (Table 3). By conserving soil moisture, it allows plants to maintain photosynthesis for longer periods under drought stress, thus alleviating its negative impacts [59,64,65,66,67,68]. The Duke FACE experiment, for instance, showed that while the absolute CO_2_-driven growth enhancement was greatest in wet years, the relative benefit of elevated CO_2_ was most pronounced during dry periods [43,81,82,83,88]. Conversely, in systems where water is not a primary limiting factor (e.g., in some wet temperate or light-limited forests), the advantages conferred by increased WUE are marginal.

Similarly, other climate factors interact with CO_2_ in a non-linear fashion. Rising temperatures, for example, may extend the growing season in temperature-limited boreal forests, potentially unlocking some CO_2_ benefits [43,70,89,90,91]. However, in tropical regions, warming can push forests beyond their thermal optima, inducing heat stress that can negate or even reverse any positive CO_2_-driven gains [33,37,71,92,93,94]. Furthermore, the allocation of the additionally assimilated carbon is a critical internal modulator. Plants often respond to elevated CO_2_ by increasing the proportion of carbon allocated belowground to roots and mycorrhizal fungi [52,53,72,95]. This can be interpreted as an adaptive strategy to “invest” the extra carbon in foraging for limiting nutrients like nitrogen and phosphorus, thereby attempting to alleviate the very bottleneck discussed in the previous section [73,96,97,98]. However, this comes at a trade-off: increased belowground investment may occur at the expense of aboveground woody biomass production, the component most often measured as ecosystem productivity. Incorporating rhizosphere processes into Earth System Models (ESMs) remains a major challenge, as uncertainties persist in parameterizing root–microbe interactions, nutrient–water coupling, and the feedbacks between belowground carbon allocation and nutrient cycling. These allocation shifts also have complex and still poorly understood consequences for long-term soil carbon storage, mediated through changes in root exudation and microbial community dynamics [74,75,77,99,100,101,102].

In summary, the realized effect of elevated CO_2_ on any given forest is not a fixed property, but an emergent outcome of its interaction with the local hydrology, climate, and the adaptive allocation strategies of the plants themselves.

**Table 3:** Water Use Efficiency and Hydrological Responses.

Response	Description	Magnitude	Key References
Stomatal conductance reduction	Decreased stomatal opening under elevated CO_2_	10–30%	[9,59]
Leaf-level WUE increase	Increased carbon fixed per unit water transpired	30–60%	[61]
Canopy-level transpiration	Changes in whole-canopy water use, often less than expected from leaf-level changes	0–20%	[62,63]
Soil moisture effects	Increased soil moisture due to reduced transpiration	5–15%	[64,65]
Drought interaction	Enhanced CO_2_ effects during drought periods	Variable	[58,66]
Runoff and streamflow	Changes in watershed hydrology due to altered transpiration	0–10%	[67,68]
Regional water cycling	Broader hydrological cycle impacts	Complex	[62,65]

Note: This table details the mechanisms, magnitudes, and key references for the effects of elevated CO_2_ on water use and the hydrological cycle at various scales, primarily through its influence on plant stomatal behavior. These data provide specific quantitative support for the argument in the main text that Water Use Efficiency (WUE) acts as a critical dynamic modulator.

## 6. Experimental Approaches and Evidence

The evidence synthesized in this review paints a clear picture: the constrained response of forests to elevated CO_2_ is an emergent property of complex, cross-scale interactions [103,104,105]. We have deconstructed the journey of the CO_2_ molecule from its entry into the leaf to its ultimate fate within the ecosystem, revealing a hierarchy of constraints [106,107,108]. The large biochemical potential for photosynthetic enhancement is first attenuated by near-universal plant-level acclimation [109,110,111,112,113]. This remaining potential then confronts the unyielding bottleneck of ecosystem nutrient availability, which in many cases proves to be the dominant limiting factor [112]. Finally, the realized effect is dynamically modulated by the interplay of water availability, climate variables, and internal carbon allocation strategies [114]. This integrated understanding, moving beyond a simple fertilization paradigm, is essential for accurately assessing the future of the global carbon cycle.

**Figure 4:**
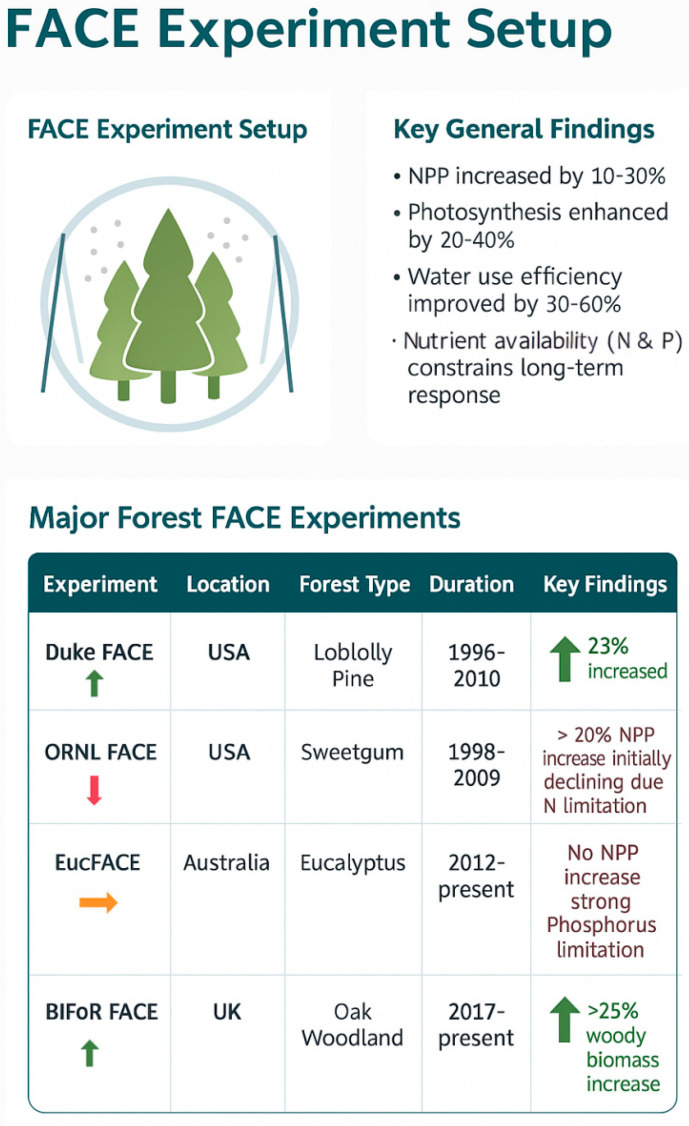
Major forest FACE (Free-Air CO_2_ Enrichment) experiments and their general findings. The upper panel illustrates the FACE setup and summarizes overall responses: net primary production (NPP) increased by 10–30%, photosynthesis enhanced by 20–40%, water-use efficiency improved by 30–60%, and nutrient availability (N and P) constrains long-term responses. The lower panel lists major FACE sites (Duke FACE, ORNL FACE, EucFACE, BIFoR FACE) with their key findings. Arrow colors indicate experimental outcomes: green = positive increase, red = decline due to nutrient limitation, orange = no significant increase because of phosphorus limitation.

Our current understanding has been built upon a foundation of diverse experimental approaches, with Free-Air CO_2_ Enrichment (FACE) experiments standing as the “gold standard” for examining ecosystem responses under realistic field conditions [13,14]. Figure 4 provides a schematic overview of the FACE methodology and summarizes its key general findings. As further detailed in Table 4, these invaluable, long-term experiments have been instrumental in moving the field beyond simplistic leaf-level predictions. They forced the scientific community to confront the complex realities of nutrient limitation—most starkly demonstrated by the contrasting results of the Duke and EucFACE experiments [42,54]—and the critical role of hydrological feedbacks [17,61]. However, the high cost and logistical complexity of FACE experiments mean they are few in number and unevenly distributed globally, with a critical lack of representation in tropical biomes, which remains a major knowledge gap [106,107,109]. Other approaches, such as open-top chambers and retrospective tree-ring analyses, provide complementary insights, but FACE experiments remain our most important anchor to ecosystem reality [58,79,99,101].As illustrated in Figure 4, the outcomes of major FACE experiments consistently show initial enhancements of photosynthesis, NPP, and water-use efficiency under elevated CO_2_, but with clear nutrient-based constraints that differ among sites and forest types.

Mechanistic models are indispensable for scaling these experimental insights across space and time and for projecting future forest dynamics under various climate scenarios [56,84,111,112,113]. As outlined in Table 5, a range of modeling approaches exists, from detailed leaf-level models to globe-spanning Earth System Models. Yet, current-generation models still struggle to accurately represent the key constraints discussed in this review [75,98,114,115,116]. A growing body of evidence shows that many models tend to overestimate the CO_2_ fertilization effect precisely because their representations of nutrient cycling—particularly phosphorus limitation—remain overly simplistic or are absent altogether [52,76,117,118,119,120]. This limitation in our primary predictive tools represents a major source of uncertainty in projecting the future of the terrestrial carbon sink.

This critical gap between our synthesized understanding of processes and our current predictive capability defines a clear path forward for the research community. Table 6 summarizes the key research priorities needed to bridge these gaps. To reduce uncertainty and improve our forest management strategies, future research must prioritize the following:

1. Multi-Factor Experimental Platforms: The era of single-factor manipulation is reaching its limits. The most pressing need is for next-generation experiments that manipulate CO_2_ in concert with other critical global change drivers, especially warming and altered precipitation regimes, to understand their crucial synergistic and antagonistic effects [22,77,100,121].
2. Targeted Research in Underrepresented Ecosystems: Given their outsized role in the global carbon cycle, establishing and sustaining long-term, multi-factorial experiments in tropical forests is arguably the single most important empirical priority [55,76,107].
3. Next-Generation Model Development: A concerted effort is required to integrate robust and interacting nutrient cycles (N and P) and sophisticated plant hydraulic modules into ESMs [37,74,81]. Crucially, a stronger culture of data-model fusion is needed to parameterize, test, and constrain these models with experimental data [56,78,122,123].
4. Focus on Belowground Processes: The intricate interactions within the rhizosphere—involving roots, mycorrhizal fungi, and microbial communities—mediate nutrient uptake and carbon storage. Deeper investigation into these belowground processes is fundamental to understanding the long-term stability of ecosystem responses [73,82,124].

Although FACE experiments are widely regarded as the ecosystem-scale “gold standard,” their representativeness is constrained by several important factors. First, FACE sites are unevenly distributed across the globe, with a critical lack of experiments in tropical forests, which are among the most influential biomes for the global carbon cycle. Second, many FACE studies are conducted in relatively simple or young plantations, which may not capture the structural and functional complexity of old-growth or highly diverse forests. Third, the relatively short duration of most FACE experiments and their logistical and financial constraints limit their ability to fully represent long-term and large-scale ecosystem responses. These limitations underscore the need for establishing long-term, multi-factorial experiments in underrepresented tropical ecosystems and for coupling FACE results with data–model integration approaches to reduce uncertainties when extrapolating to the global scale [98,118,120,125].

## 7. Conclusions and Implications

The response of forest photosynthesis to rising atmospheric CO_2_ is far more nuanced and constrained than a simple “fertilization effect” would suggest. This review has reframed the issue, presenting elevated CO_2_ as a complex modulator whose effects are systematically attenuated by a hierarchy of constraints, from photosynthetic acclimation at the leaf level to overriding nutrient limitations at the ecosystem scale [58,79,110,111,112,113]. The variability in forest responses across biomes is not random, but a predictable consequence of the interplay between CO_2_ and the local context of nutrient availability, water status, and climate. Recognizing that nutrient cycles—not carbon availability—are often the ultimate arbiters of long-term forest productivity in a high-CO_2_ world is a critical paradigm shift. This synthesized understanding has profound implications for both science and practice [114].

### 7.1. Implications for Forest Management and Conservation

Our findings challenge the passive assumption that forests will uniformly benefit from rising CO_2_. Instead, proactive and context-specific management strategies are required. For instance, in temperate forests where nitrogen is the primary constraint, management practices that enhance nitrogen availability (e.g., selection of N-fixing species in mixed plantations) may be necessary to unlock any potential CO_2_ benefit [45,49]. Conversely, in phosphorus-limited tropical regions, afforestation or restoration efforts might gain little productivity boost from rising CO_2_, and conservation priorities should focus on preserving existing carbon stocks and biodiversity rather than banking on enhanced growth [54,76,107]. Furthermore, the amplified benefit of CO_2_ under drought suggests that in water-limited regions, thinning practices that reduce water competition could synergistically enhance forest resilience and productivity in a future climate [59,67,104,108].

### 7.2. Implications for Climate Change Mitigation

The constrained CO_2_ fertilization effect implies that the capacity of forests to act as a sustained carbon sink, thereby mitigating climate change, may be overestimated in many current Earth System Models [52,75,107]. The strong evidence for nutrient limitation suggests that the terrestrial carbon sink is not infinite and may saturate sooner than previously thought, placing a greater urgency on direct emissions reduction policies [48]. Reforestation and afforestation remain vital natural climate solutions, but their effectiveness must be evaluated through the lens of nutrient availability [93,94]. Projects targeted at fertile soils are likely to yield far greater carbon sequestration benefits per unit area than those on nutrient-poor lands. Therefore, integrating high-resolution soil nutrient maps into global climate solution planning is essential for maximizing their impact and cost-effectiveness.

In closing, as atmospheric CO_2_ continues to rise, our ability to predict and manage forest responses depends on embracing this complexity. By shifting our research focus from single-factor effects to multi-stressor interactions [22,77,118,125], and by improving our models to reflect the fundamental constraints of nutrient cycling [37,56,78], we can develop more robust and effective strategies for stewarding the world’s forests through the 21st century. The hierarchical framework presented here not only integrates the classical concepts of PNL and source–sink imbalance but also establishes a testable cross-scale causal chain, providing theoretical support for model improvement and the design of future multifactor experiments [102,114].

## Figures and Tables

**Table 1:** Key Mechanisms and Detailed Descriptions of Photosynthetic Acclimation.

Mechanism	Description	Key References
Rubisco content reduction	Decreased Rubisco content and activity (Vcmax) after long-term CO_2_ exposure	[9,33]
Electron transport adjustment	Changes in maximum electron transport rate (Jmax) and Jmax/Vcmax ratio	[37,38]
Carbohydrate accumulation	Increased leaf starch and soluble sugar content leading to feedback inhibition	[11]
Nitrogen reallocation	Shift in nitrogen allocation from Rubisco to light harvesting components	[35,36]
Source-sink regulation	Imbalance between carbohydrate production and utilization/export	[17]
Gene expression changes	Downregulation of genes related to photosynthesis and carbon fixation	[33]
Morphological adaptations	Changes in leaf thickness, stomatal density, and chloroplast structure	[11]

Note: This table provides a detailed list of the primary physiological and molecular mechanisms involved in photosynthetic acclimation by plants in response to elevated CO_2_. Together, these mechanisms constitute the first intrinsic feedback loop that attenuates the long-term stimulatory effect of CO_2_, providing specific mechanistic support for the “first brake” discussed in the main text.

**Table 4:** Major Forest FACE Experiments and Their Key Findings in Revealing Ecosystem Constraints.

FACE Experiment	Location	Forest Type	Duration	Key Findings	Reference
Duke FACE	USA	Loblolly Pine	1996–2010	23% increase in NPP; 40% increase in WUE; Stronger response in wet years	[17]
Oak Ridge FACE	USA	Sweetgum	1998–2009	20% increase in NPP initially, declining to 10% over time; Progressive nitrogen limitation	[49]
EucFACE	Australia	Eucalyptus	2012–present	No significant increase in NPP; Phosphorus limitation; Enhanced WUE	[52,53]
BIFoR FACE	UK	Oak Woodland	2017–present	25% increase in woody biomass; Sustained photosynthetic enhancement	[89,90]
Hofstetten FACE	Switzerland	Mixed Deciduous	2009–2015	Weak growth response in mature trees; Significant understory response	[18]
AspenFACE	USA	Aspen, Birch, Maple	1997–2009	Species-specific responses; Interactions with O3; Altered competitive dynamics	[79]
Amazon FACE	Brazil	Tropical Rainforest	2016–present	Early results show complex responses; Phosphorus limitation important	[54]

Note: This table summarizes the most influential forest FACE experiments, providing the core ecosystem-scale evidence that underpins the arguments of this review.

**Table 5:** Major Modeling Approaches: Strengths and Limitations within the Argumentative Framework.

Model Type	Description	Strengths	Limitations	Key References
Leaf-level biochemical	Models of photosynthetic processes based on Farquhar equations	Mechanistic understanding of CO_2_ effects	Limited scaling to ecosystem level	[23,85]
Ecosystem models	Simulate carbon, water, and nutrient cycling in forest ecosystems	Integration of multiple processes	Parameter uncertainty, simplifications	[74,86]
Dynamic global vegetation models	Global-scale models of vegetation dynamics and biogeochemistry	Large spatial coverage, vegetation dynamics	Coarse resolution, process simplification	[13]
Earth system models	Coupled models of atmosphere, ocean, land, and ice	Integration of climate feedbacks	Computational demands, uncertainty propagation	[3,7,87]
Data-model fusion	Integration of observations with models through data assimilation	Improved parameter constraints, uncertainty quantification	Data limitations, computational complexity	[76,77]
Machine learning approaches	Statistical models trained on observational data	Capture complex patterns without prior assumptions	Limited mechanistic insight, extrapolation issues	[80,84]

Note: This table outlines the various types of models used to predict forest responses, highlighting their strengths and, crucially, their limitations in capturing the constraints emphasized in this review.

**Table 6:** Research priorities and approaches for advancing understanding of forest photosynthetic responses to elevated CO_2_ in a multi-stressor world.

Research Area	Key Questions/Approaches/Key References
Multi-factor interactions	Key Questions: How do elevated CO_2_ effects interact with warming, drought, and ozone? Approaches: Multifactor manipulations, climate-gradient transects, model ensembles. Key References: [33,34,61,62,109,119].
Nutrient–hydrology linkages	Key Questions: How do nutrient cycling and water availability jointly regulate CO_2_ responses? Approaches: FACE × irrigation/fertilization factorials, isotope tracers. Key References: [52,53].
Belowground processes	Key Questions: What role do roots, mycorrhizae, and microbial communities play in modulating long-term CO_2_ responses? Approaches: Root exclusion, mycorrhizal manipulation, metagenomics. Key References: [81,86].

Note: This table is the constructive centerpiece of the review. It identifies critical knowledge gaps and proposes specific, actionable research priorities, directly addressing the need for a “constructive viewpoint.”

## Data Availability

Data is contained within the article.
